# Berberine and Cisplatin Exhibit Synergistic Anticancer Effects on Osteosarcoma MG-63 Cells by Inhibiting the MAPK Pathway

**DOI:** 10.3390/molecules26061666

**Published:** 2021-03-17

**Authors:** Xianxian Gao, Chen Zhang, Yanjie Wang, Ping Zhang, Jingyu Zhang, Tie Hong

**Affiliations:** Department of Pharmacology, School of Pharmaceutical Sciences, Jilin University, Changchun 130021, China; gaoxx18@mails.jlu.edu.cn (X.G.); zhangchen18@mails.jlu.edu.cn (C.Z.); yjwang19@mails.jlu.edu.cn (Y.W.); zhangping19@mails.jlu.edu.cn (P.Z.); jingyu20@mails.jlu.edu.cn (J.Z.)

**Keywords:** berberine, cisplatin, MG-63, combined treatment, apoptosis

## Abstract

Berberine (BBR) has been reported to have potent anticancer activity and can increase the anticancer effects of chemotherapy drugs. The present study aims to investigate whether BBR and cisplatin (DDP) exert synergistic effects on the osteosarcoma (OS) MG-63 cell line. In the present study, MG-63 cells were treated with BBR and DDP alone or in combination. The effects of these therapeutics on cell viability, colony formation, migration, invasion, nuclear morphology, apoptosis, and the cell cycle, as well as their role in regulating the expression of proteins related to apoptosis, the cell cycle, and the mitogen-activated protein kinase (MAPK) pathway, were determined. The results demonstrated that BBR or DDP significantly inhibited the proliferation of MG-63 cells in a dose- and time-dependent manner. The combination treatment of BBR and DDP exerted a prominent inhibitory effect on proliferation and colony formation. Furthermore, the results showed that the combination treatment of BBR and DDP enhanced the inhibition of cell migration and invasion and reversed the changes in nuclear morphology. The results showed that the combination treatment of BBR and DDP induced apoptosis and cell cycle arrest in the G0/G1 phase. Mechanistically, the combination treatment of BBR and DDP inhibited the expression of MMP-2/9, Bcl-2, CyclinD1, and CDK4, enhanced the expression of Bax and regulated the activity of the MAPK pathway. Collectively, our data suggest that the combination therapy of BBR and DDP markedly enhanced OS cell death.

## 1. Introduction

Osteosarcoma (OS) is a common malignant tumor that originates from the stroma of osteogenic material [1]. OS mainly occurs in the bones of children and adolescents and has a high propensity for local invasion and early systemic metastasis [2]. At present, treatment usually includes neoadjuvant chemotherapy, surgical resection, and a successive course of chemotherapy following surgery [3]. Although prognosis has improved for patients with localized disease, patients with metastatic disease still have a poor prognosis [4]. Patients with metastatic disease at diagnosis or with recurrent disease have a five-year survival rate of only 20% [5].

Cisplatin (DDP), a first-line chemotherapeutic drug, is widely used to treat various tumors, including bladder cancer, cervical cancer, small cell lung cancer, and gastric cancer [6]. As one of the crucial drugs in OS chemotherapy [7], DDP exerts potent anti-OS activity, but its application is limited by drug resistance and side effects, including genotoxicity, nephrotoxicity, and acute myelotoxicity [8,9]. A higher cumulative dose and higher doses per treatment of DDP result in greater irreversible kidney injury [10,11,12]. Hence, it is necessary to establish a more effective and safe treatment strategy that combines a low dose of DDP with other complementary agents to overcome drug resistance and reduce toxicity.

Berberine (BBR) is an isoquinoline-derived alkaloid that has been widely used in the clinic owing to its multiple biochemical and pharmacological effects [13,14]. BBR has been extensively used in the clinic for the treatment of various diseases due to its antibacterial, anti-inflammatory, antidiabetic, and cardioprotective capabilities [15,16,17]. Additionally, it has been shown that BBR may inhibit the growth of a variety of human cancer cell lines, including prostate cancer [18,19], colon cancer [20], lung cancer [21], nasopharyngeal cancer [22], and breast cancer [23,24] cell lines. BBR may also induce the apoptosis of human osteosarcoma cells via the mitochondrial pathway of apoptosis [25,26]. Recent studies have also shown that BBR can increase the sensitivity of cancer cells to radiation and chemotherapy [27,28,29]. Therefore, combinatorial treatments, including a combination of BBR with DDP, may help improve the efficacy of chemotherapy.

We examined whether BBR synergistically potentiated the anticancer activity of DDP in osteosarcoma MG-63 cells. We also evaluated the possible molecular signaling pathway underlying this effect.

## 2. Results

### 2.1. Effects of BBR Alone, DDP Alone, and Their Combination on the Viability of MG-63 Cells

**Figure 1:**
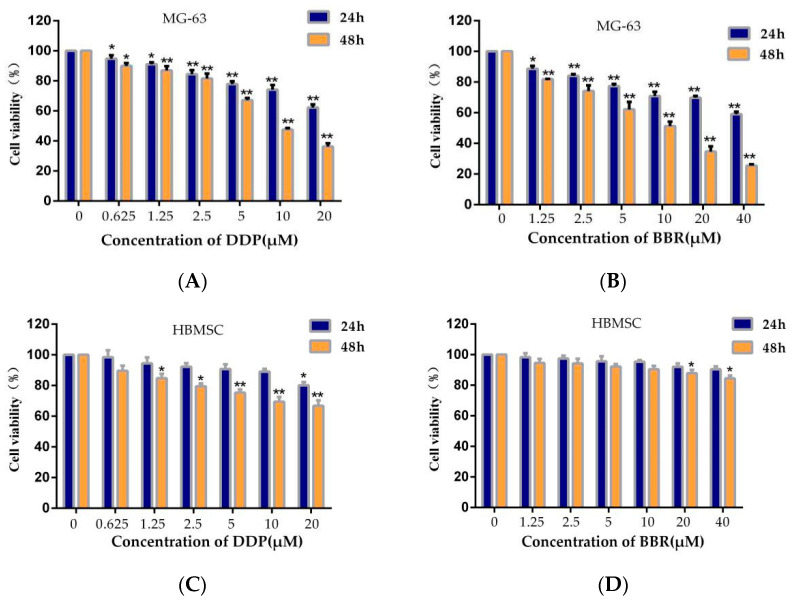

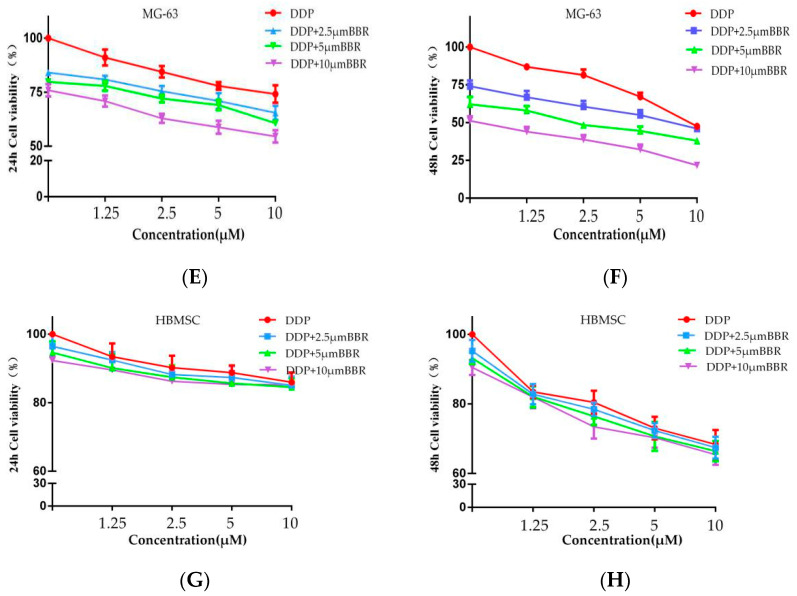
Effects of berberine (BBR) and/or cisplatin (DDP) on MG-63 and HBMSC cell viability**.** (**A**) Viability of MG-63 cells after treatment with different concentrations of DDP for 24 or 48 h. (**B**) Viability of MG-63 cells after treatment with different concentrations of BBR for 24 or 48 h. (**C**) Viability of HBMSC cells after treatment with different concentrations of DDP for 24 or 48 h. (**D**) Viability of HBMSC cells after treatment with different concentrations of BBR for 24 or 48 h. (**E**) Viability of MG-63 cells after treatment with BBR and/or DDP for 24 h. (**F**) Viability of MG-63 cells after treatment with BBR and/or DDP for 48 h. (**G**) Viability of HBMSC cells after treatment with BBR and/or DDP for 24 h. (**H**) Viability of HBMSC cells after treatment with BBR and/or DDP for 48 h. The data are presented as the mean ± SD of three separate experiments; * *p* < 0.05 ** *p* < 0.01, compared with the control group.

First, we analyzed the effects of different concentrations of BBR and DDP on MG-63 and HBMSC cell viability using MTT assays. The results showed that BBR or DDP exhibited a time- and dose-dependent inhibitory effect on MG-63 cells (Figure 1A,B). After 24 h of treatment, the 50% inhibitory concentration (IC50) of BBR was 77.08 μM in MG-63 cells, and the IC50 was 12.42 μM after 48 h treatment. In addition, at 24 and 48 h, the IC50 values of DDP were 94.74 and 9.62 μM in MG-63 cells, respectively. However, BBR had no change on HBMSC cell viability (Figure 1D). Our study also showed that DDP had mild cytotoxicity on HBMSC cells, the IC50 value of DDP for HBMSC cells was 236.60 μM for 24 h and 89.65 μM for 48 h (Figure 1C).

To determine whether BBR enhances the effect of DDP, we analyzed the viability of MG-63 cells and HBMSC cells treated with different concentrations of BBR (2.5, 5, or 10 μM) in combination with DDP (1.25, 2.5, 5, or 10 μM) for 24 or 48 h to explore the effects of the combination. As shown in Figure 1E,F, compared to DDP alone, the combination of BBR and DDP induced significantly higher cytotoxicity in the MG-63 cells. However, BBR and DDP had no exhibit synergistic toxicity for HBMSC cells (Figure 1G,H). Furthermore, Chou–Talalay analysis [30] was used to calculate the combination index (CI) of BBR and DDP for the MG-63 cells. The results are shown in Table 1, and the vast majority of combinations showed synergistic effects (CI < 1). CI values below 1 indicate that the drugs had a synergistic effect.

BBR (5 μM) and DDP (2.5 μM) caused approximately 50% inhibition of MG-63 cell growth, and CI < 1, indicating that the drugs had a synergistic effect at this concentration. Therefore, in the following study, cotreatment with BBR (5 μM) and DDP (2.5 μM) was used.

### 2.2. Cotreatment with BBR and DDP Synergistically Inhibited the Migration and Invasion of MG-63 Cells

**Figure 2:**
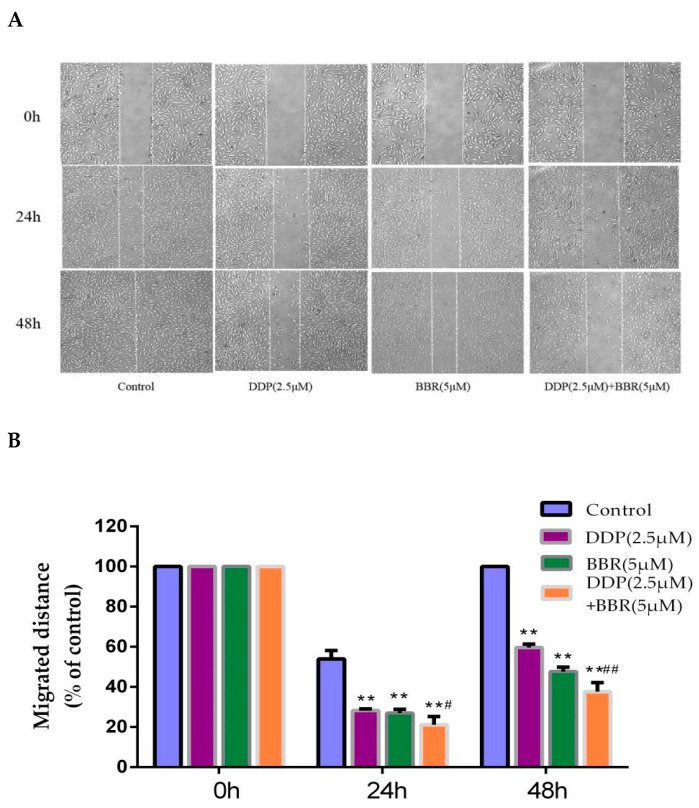

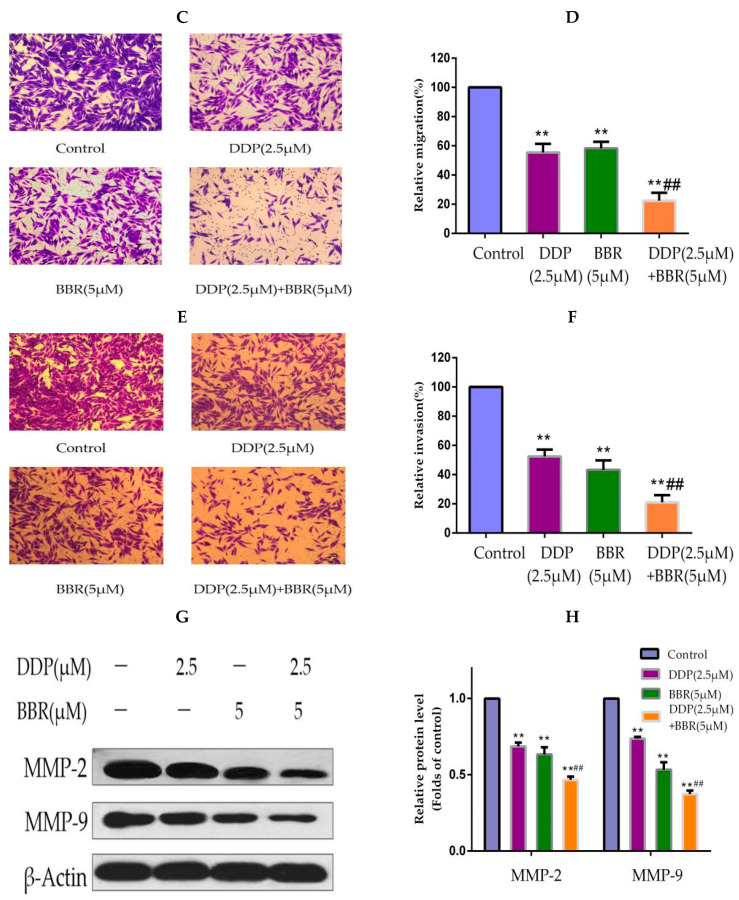
BBR and DDP suppress the migration and invasion capacity of MG-63 cells**.** (**A**,**B**) Micrographs of wound-healing assays with MG-63 cells treated with BBR and/or DDP. Images were obtained at 0, 24, and 48 h (100× magnification). (**C**,**D**) A transwell assay was used to detect the migration of MG-63 cells. The number of migratory cells was observed and counted by using a light microscope (100× magnification). (**E**,**F**) A transwell assay was used to detect the invasion of MG-63 cells. The number of invasive cells was observed and counted by using a light microscope (100× magnification). (**G**,**H**) Western blot analysis of MMP-2 and MMP-9 in MG-63 cells. The data are presented as the mean ± SD of three independent experiments; ** *p* < 0.01, compared with the control group. ^##^ *p* < 0.01, compared with the monotherapy group.

A wound-healing assay was performed to evaluate the effects of the combination treatment of BBR and DDP on MG-63 cells. The results indicated that the cells in medium displayed a higher rate of migration into the scratched wound area relative to drug-treated cells. A moderate inhibition of migration was detected in the cancer cells treated with either BBR or DDP, whereas a significant inhibition of migration was observed in the cells cotreated with BBR and DDP (Figure 2A,B).

Transwell assays were used to determine the effects of the combination treatment of BBR and DDP on MG-63 cell migration and invasion. Treatment with BBR or DDP alone suppressed cell migration and invasion; however, combined treatment significantly enhanced this inhibition (Figure 2C–F).

To determine the detailed mechanism underlying the potential effect of the combined treatment on cell migration and invasion, we used Western blotting analysis to determine the levels of key protein markers (MMP2/9). These results confirmed that BBR enhances the DDP-mediated inhibition of MG-63 cell migration and invasion (Figure 2G,H).

### 2.3. Cotreatment with BBR and DDP Synergistically Inhibited the Cloning Ability of MG-63 Cells

**Figure 3:**
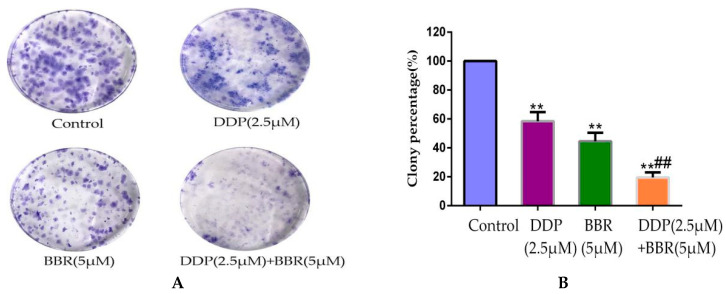
BBR and DDP inhibited the cloning Ability of MG-63 cells. (**A**) Representative images showing the effect of BBR and/or DDP on the loss of colony formation ability in MG-63 cells post-treatment. (**B**) Bars (mean ± SD; *n* = 3) represent the relative percentage of colony formation ability in MG-63 cells post-treatment. The data are presented as the mean ± SD of three independent experiments; ** *p* < 0.01, compared with the control group. ^##^ *p* < 0.01, compared with the monotherapy group.

We performed colony formation assays to analyze whether the combination of BBR and DDP resulted in a synergistic loss of clonogenicity in MG-63 cells. As shown in Figure 3A,B, a significant loss (70–80%) in the colony formation ability was observed in BBR- and DDP-treated MG-63 cells compared to that in cells treated with either BBR (≈50%) or DDP (≈40%) alone. These observations further confirmed the synergistic effect of the BBR and DDP combination treatment.

### 2.4. Cotreatment with BBR and DDP Synergistically Induced the Apoptosis of MG-63 Cells

**Figure 4:**
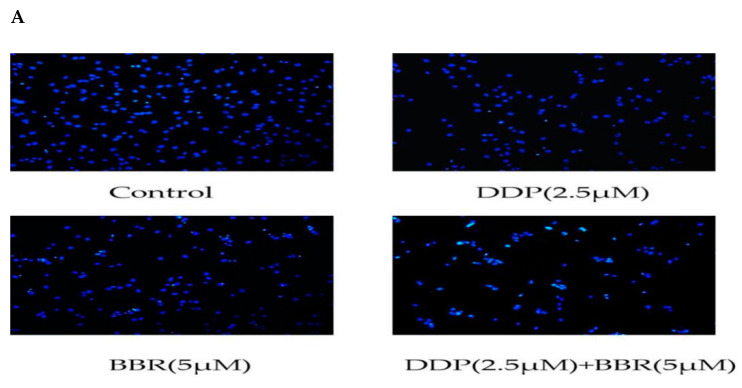

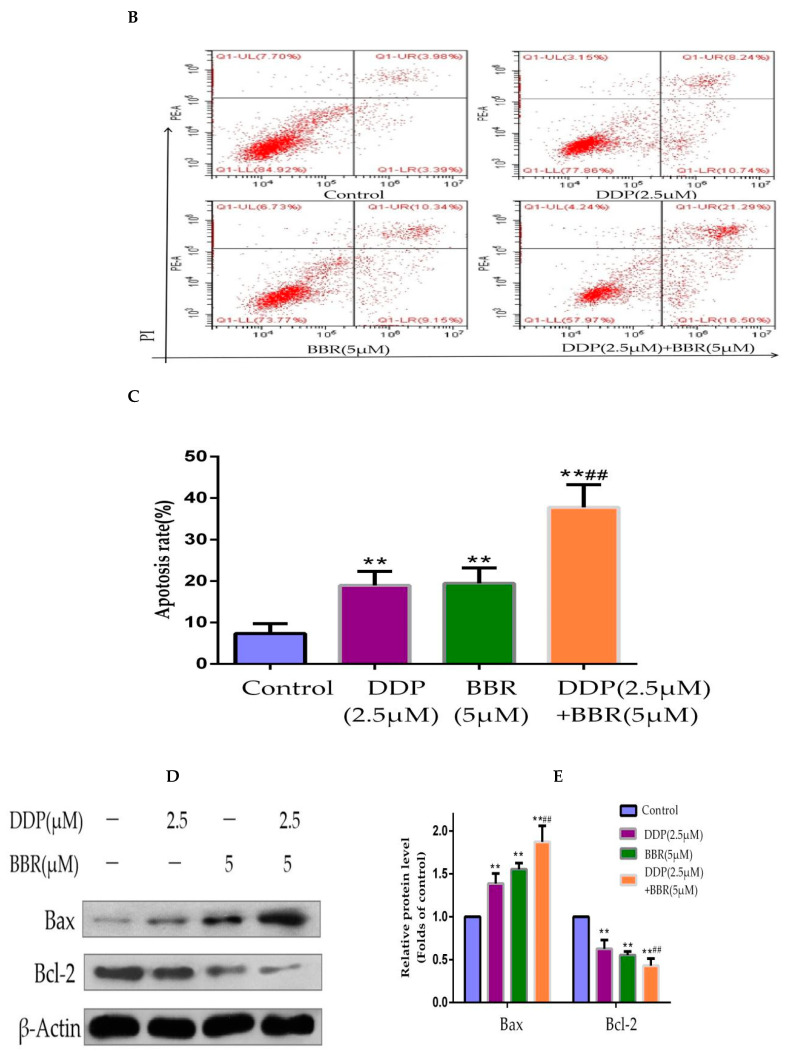
Effect of BBR and DDP alone and in combination on apoptosis**.** (**A**) Hoechst 33258 staining of MG-63 cells treated with BBR and/or DDP for 48 h. Apoptotic cells were identified by the presence of bright-blue fluorescence and highly condensed or fragmented nuclei (100× magnification). (**B**,**C**) The apoptosis of MG-63 cells was determined by flow cytometry after staining with annexin V-FITC/P. (**D**,**E**) The levels of cleaved Bcl-2 (antiapoptotic protein) and Bax (proapoptotic protein) were detected by Western blotting. The data are presented as the mean ± SD of three independent experiments; ** *p* < 0.01, compared with the control group. ^##^ *p* < 0.01, compared with the monotherapy group.

Hoechst staining was used to assess the nuclear morphological changes in MG-63 cells using a fluorescence microscope. The results from Hoechst staining showed (Figure 4A) that the control group had round nuclei that were homogeneously stained blue. In cases of cell death, markedly induced chromatin condensation or fragmentation is shown in bright blue. Cells treated with BBR and DDP showed much more bright-blue fluorescence and condensed nuclei than untreated cells and cells treated with BBR or DDP alone.

Induction of apoptosis is a key mechanism through which anticancer compounds exert their effects. Therefore, we investigated whether the cytotoxicity of BBR and DDP was associated with the induction of apoptosis in MG-63 cells. As shown in Figure 4B,C, the flow cytometry assay results demonstrated that BBR and DDP could both induce MG-63 cell apoptosis. The apoptotic rate in the cells treated with the combination of BBR and DDP was markedly higher than that in the control-treated cells and the cells treated with either drug alone.

To further characterize apoptosis, Western blotting was performed. We tested the expression of the proapoptotic protein Bax and the antiapoptotic protein Bcl-2 in MG-63 cells. The results showed that the protein level of Bax in the combined group was higher than that in the monotherapy and control groups. Conversely, the protein level of Bcl-2 in the combination group was the lowest (Figure 4D,E).

### 2.5. Cotreatment with BBR and DDP Synergistically Arrested the Cell Cycle of MG-63 Cells

**Figure 5:**
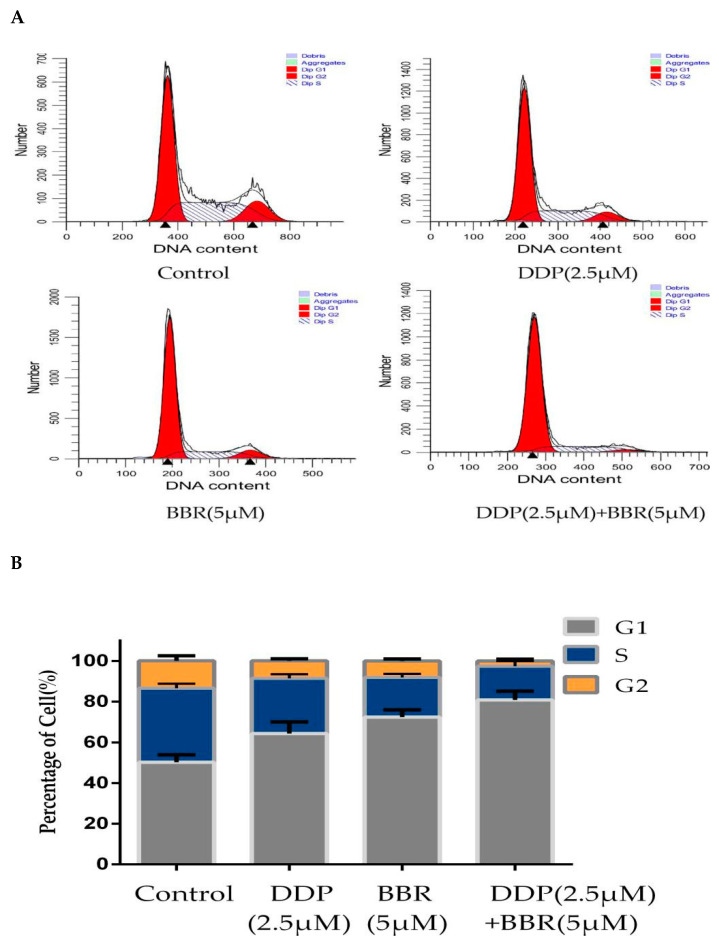

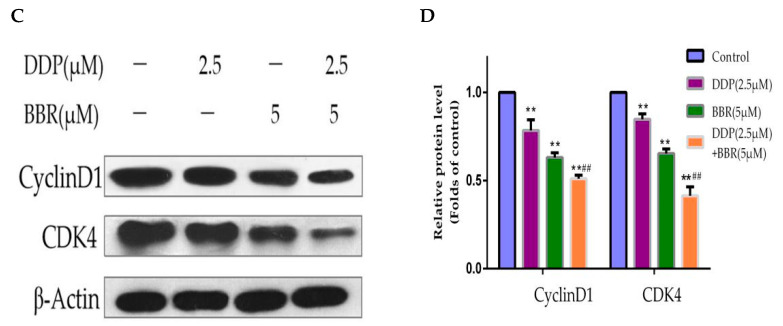
Effect of BBR and DDP alone and in combination on the cell cycle. (**A**) MG-63 cells treated with BBR and/or DDP for 48 h. The cell cycle was evaluated by flow cytometry using PI staining. (**B**) Percentage of cells distributed in each phase of the cell cycle. (**C**,**D**) The levels of cleaved CyclinD1 and CDK4 were detected by Western blotting. The data are presented as the mean ± SD of three independent experiments; ** *p* < 0.01, compared with the control group. ^##^ *p* < 0.01, compared with the monotherapy group.

To investigate the effects of BBR and DDP on the cell cycle, MG-63 cells were incubated with BBR and/or DDP for 48 h, and the cell cycle distribution was analyzed by flow cytometry. The results indicated that BBR or DDP significantly increased the number of MG-63 cells in the G0/G1 phase compared with the control, and this effect was even enhanced in the BBR and DDP cotreatment groups compared with the single treatment groups (Figure 5A,B). To explore the potential molecular mechanism underlying G0/G1 cell cycle arrest, the expression levels of cell-cycle-related proteins were determined by Western blotting analysis. As shown in Figure 5C,D, the expression levels of CyclinD1 and CDK4 were decreased after either BBR or DDP treatment alone, while the combination treatment further reduced the CyclinD1 and CDK4 expression in MG-63 cells.

### 2.6. Mitogen-Activated Protein Kinase (MAPK) Pathway Participates in the Synergistic Effects of the Combined Treatment

**Figure 6:**
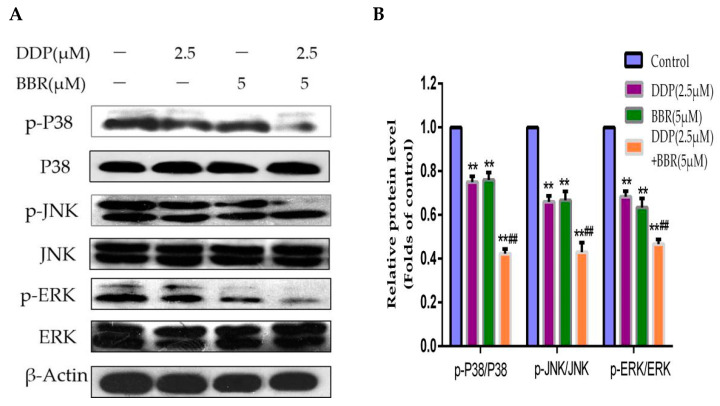
Effect of BBR and DDP alone and in combination on MAPK signaling. (**A**,**B**) Cells were treated with BBR or/and DPP for 48 h. The levels of cleaved MAPK were detected by Western blotting. The data are presented as the mean ± SD of three independent experiments; ** *p* < 0.01, compared with the control group. ^##^ *p* < 0.01, compared with the monotherapy group.

Since the mitogen-activated protein kinase (MAPK) pathway is significantly associated with neoplastic transformation and plays an important role in regulating cellular apoptosis, we investigated the effect of BBR and DDP on MAPK pathway-related proteins. We evaluated the protein expression levels of p-P38, P38, p-JNK, JNK, p-ERK, and ERK by Western blotting analysis (Figure 6A,B). Our results demonstrated that compared with the control, single-drug treatment downregulated the expression of p-P38, p-JNK, and p-ERK, and these proteins were further markedly downregulated by the combined treatment of BBR and DDP.

## 3. Discussion

For the majority of patients with osteosarcoma, it is difficult to select the optimum therapeutic regimens. Compared with noncisplatin regimens, cisplatin-based chemotherapy has achieved considerable success in improving the prognosis and five-year survival rate of patients [31]. However, tumor cells are able to repair damage, evade apoptosis, and return to their originally high proliferation rates after treatment with low doses of DDP, and chemotherapy with DDP is effective only at high doses [32]. Furthermore, long-term exposure to a high dose of DDP leads to the development of drug resistance in tumor cells, limiting its clinical success in cancer chemotherapy [33]. Recently, the combination of naturally occurring compounds with conventional chemotherapeutic drugs has gained attention [34]. Previous studies have reported that BBR can enhance the therapeutic effect of DDP in ovarian cancer cells [35] by inducing necroptosis and apoptosis. However, the effects of BBR on cisplatin-treated osteosarcoma cells have not been elucidated to date.

In the present study, we used BBR (5 μM) and DDP (2.5 μM) for our experiments. Collectively, the results show that the combination of BBR and DDP had significant effects on apoptosis induction, cell cycle arrest, and cell invasion inhibition in MG-63 cells compared with the individual and control treatments. Moreover, cotreatment with BBR and DDP synergistically regulated the activity of the MAPK pathway. These results suggested that the combination of BBR and DDP has strong inhibitory effects on OS cells.

It is well known that the inhibition of proliferation is closely associated with apoptosis. Apoptosis plays a vital role in eliminating cancer cells. Therefore, apoptosis has become the key indicator in most cancer treatments [36]. It has been reported that BBR can induce apoptosis in various cancer cells [37]. We also confirmed by annexin V-FITC/propidium iodide (PI) staining that BBR combined with DDP can significantly increase the number of apoptotic cells. Moreover, the expression of the apoptotic protein Bax, which is involved in the apoptotic pathway, was upregulated. The antiapoptotic protein Bcl-2 was downregulated.

During the early metastasis of osteosarcoma, MMPs play a major role in degrading the extracellular matrix, thus allowing tumor cells to migrate and accelerating metastatic progression [38]. A previous study showed that BBR inhibits cell proliferation and promotes apoptosis of non-small-cell lung cancer via the suppression of the MMP-2 signaling pathways [39]. Similarly, we observed that BBR combined with DDP significantly suppressed the migration and invasion of MG-63 cells by downregulating MMP-2 and MMP-9 expression.

The MAPK pathway is considered to be the most significant inducer of cellular apoptosis in response to chemotherapeutic drugs [40]. The MAPK signaling pathway, which includes P38, ERK, and JNK, markedly influences cell proliferation, apoptosis, differentiation, and survival [41]. Previous studies have shown that BBR inhibits the growth of human gastric cancer cells through the MAPK signaling pathway [42]. On the other hand, artesunate can increase the anticancer effect of cisplatin by inhibiting the MAPK pathway [43]. Therefore, whether the apoptosis caused by BBR and DDP is related to the MAPK signaling pathway is worthy of investigation. The results showed no significant change in the expression of total P38, JNK, and ERK, but the expression levels of the phosphorylated form of these proteins were clearly decreased after treatment with BBR or DDP, especially after treatment with both drugs. This result suggested that BBR effectively enhanced the antitumor activity of DDP by inducing apoptosis through the inhibition of the MAPK signaling pathway in MG-63 cells.

## 4. Materials and Methods

### 4.1. Cell Culture

The human osteosarcoma MG-63 cell line and HBMSC cell were purchased from the National Infrastructure of Cell Line Resource (Shanghai, China). The cells were cultured in minimum essential medium (MEM) with 10% heat-inactivated FBS, penicillin (100 U/mL), and streptomycin (100 U/mL). The cells were incubated at 37 °C in a 5% CO_2_ incubator. The medium was changed every two days. Following treatment, the cells were harvested by trypsinization.

### 4.2. Drugs and Antibodies

Berberine (purity: ≥98%) and cisplatin (purity: ≥99%) were purchased from Sigma Aldrich (St. Louis, MO, USA). Anti-MMP-2, anti-MMP-9, anti-Bcl-2, anti-Bax, anti-CyclinD1, anti-CDK4, anti-JNK, anti-phospho-JNK, anti-ERK, anti-phospho-ERK, anti-P38, and anti-phospho-P38 primary antibodies were purchased from Bioss (Beijing, China). Goat antimouse IgG and goat antirabbit IgG secondary antibodies were purchased from Life Science (Santa Cruz, CA, USA).

### 4.3. Cell Viability Assay

Cells (1 × 10^5^ cells/mL) were seeded in 6-well plates overnight at 37 °C. First, the cells were exposed to DDP (0, 0.625, 1.25, 2.5, 5, 10, or 20 μM) or BBR (0, 1.25, 2.5, 5, 10, 20, or 40 μM) for 24 or 48 h. Second, to determine the combined effects of BBR and DDP, the cells were exposed to various combinations of different concentrations of BBR (2.5, 5, or 10 μM) and different concentrations of DDP (0, 1.25, 2.5, 5, or 10 μM) for 24 or 48 h. After 24 or 48 h, MTT (10 mg/mL) reagent was added, and the cells were incubated for 4 h. The formazan crystals produced in the cells were dissolved in 200 μL DMSO per well. Subsequently, the optical density was measured at 570 nm using a microplate reader.

### 4.4. Combination Index

The combined effect of DDP and BBR on the MG-63 cells was evaluated using the combination index (CI), as described previously [44]. CI analysis was performed using Calcusyn Graphing Software (Biosoft, Inc., MO, USA). Synergy was defined as CI < 1.0, antagonism as CI > 1.0, and additive effects as CI values not significantly different from 1.0.

### 4.5. Wound-Healing Assay

The cells (1 × 10^5^ cells/mL) were seeded in 6-well plates. When the cells reached 85% confluence, each well was manually scratched with 20 μL sterile pipette tips. The cells were then washed with PBS to remove the debris and cultured with complete medium containing DDP, BBR, or both at the indicated concentrations. Representative images were taken at 0, 24, and 48 h under an inverted microscope.

### 4.6. Transwell Assay

MG-63 cells were treated with DDP, BBR, or both for 48 h. Then, the cells were trypsinized, and 4 × 10^4^ cells in 200 μL serum-free MEM were transferred into the upper chamber of the transwell (8 μM pore size, BD Biosciences, St Louis, MO, USA) precoated with or without Matrigel (BD Biosciences, St Louis, MO, USA). Then, 600 μL of culture medium containing 20% FBS was added to the lower chamber. After 24 h of culture at 37 °C in a 5% CO_2_ incubator, the transwell chambers were fixed using 4% paraformaldehyde and then stained with 1% crystal violet. The cells that migrated to the lower surface of the membrane were photographed under a Leica DM2500 microscope. The average numbers of migrated cells were determined by counting three random fields (100×).

### 4.7. Cell Colony-Formation Assay

MG-63 cells were seeded in 6-well plates at a density of 500 cells per well. After 24 h of incubation, the cells were treated with different doses of the drugs for 48 h and then incubated in complete MEM media for one week. The cells were washed with PBS three times and then fixed in chilled methanol, and the colonies were stained with 0.5% crystal violet. Clones were considered to represent viable cells if they contained an excess of 50 cells.

### 4.8. Hoechst 33258 Assay

MG-63 cells were cultured in medium containing various concentrations of BBR and/or DDP for 48 h. Next, the cells were fixed with 4% polyoxymethylene, washed twice with PBS, and then incubated with 10 µg/mL Hoechst 33258 solution in the dark for 5 min at room temperature. Finally, the cells were washed three times with PBS and observed under a fluorescence microscope.

### 4.9. Flow Cytometry Analysis

MG-63 cells were cultured in 60 mm culture dishes for 24 h, and then the cells were exposed to different concentrations of BBR and/or DDP for 48 h. For the analysis of the cell cycle, the cells were washed with cold PBS, fixed with ice-cold 70% ethanol, and stored overnight at 4 °C. The cells were subsequently washed twice with PBS and incubated with 500 μL PI/RNase Staining Buffer in the dark for 15 min at room temperature. Finally, the stained cells were analyzed using a flow cytometer system (Beckman Coulter, California). For the analysis of apoptosis, the cells were washed with PBS twice and resuspended in the appropriate binding buffer. The cells were stained with annexin V-FITC (5 μL) and PI (10 mg/L) and incubated for 15 min in the dark at room temperature before analysis by flow cytometry.

### 4.10. Western Blotting Analysis

Proteins were isolated from treated MG-63 cells. The protein concentrations were measured using the BCA Protein Assay Kit. The proteins were then separated using 10% SDS-PAGE and transferred to PVDF membranes. After blocking in 5% skim milk for 2 h, the blocked membranes were incubated with primary antibodies at 4 °C overnight. The membranes were then incubated with secondary antibodies for 1 h at RT. The signals were visualized using an enhanced chemiluminescence reagent.

### 4.11. Statistical Analysis

The SPSS 20.0 statistical software package was used to perform all the statistical analyses. All the experimental values are expressed as the mean ± standard deviation (SD) of at least three independent experiments. Comparisons between groups were made by one-way analysis of variance (ANOVA), and p < 0.05 was considered significant.

## 5. Conclusions

The results of the present study indicated that BBR and DDP can inhibit proliferation, induce apoptosis and inhibit the cell cycle of MG-63 cells. Our results also suggested that the underlying mechanism of the combined therapeutic effect is by inhibiting the MAPK signaling pathway. In summary, the effects of BBR and DDP on osteosarcoma are worthy of further study.

## Figures and Tables

**Table 1:** The combination index (CI) of BBR and DDP was calculated for the MG-63 cells.

24 h						48 h			
NO.	BBR (μM)	DDP (μM)	FA *	CI	NO.	BBR (μM)	DDP (μM)	FA *	CI
**1**	2.5	1.25	0.191	0.856	**1**	2.5	1.25	0.331	0.893
**2**	2.5	2.5	0.245	0.625	**2**	2.5	2.5	0.394	0.829
**3**	2.5	5	0.290	0.628	**3**	2.5	5	0.450	0.941
**4**	2.5	10	0.346	0.729	**4**	2.5	10	0.540	1.052
**5**	5	1.25	0.221	1.132	**5**	5	1.25	0.491	0.928
**6**	5	2.5	0.279	0.769	**6**	5	2.5	0.515	0.678
**7**	5	5	0.309	0.778	**7**	5	5	0.559	0.759
**8**	5	10	0.393	0.685	**8**	5	10	0.619	0.859
**9**	10	1.25	0.291	1.126	**9**	10	1.25	0.518	0.790
**10**	10	2.5	0.371	0.668	**10**	10	2.5	0.613	0.666
**11**	10	5	0.412	0.609	**11**	10	5	0.677	0.581
**12**	10	10	0.454	0.645	**12**	10	10	0.793	0.632

* FA (fraction affected) represents the inhibitory rate of the drug on the MG-63 cells.

## Data Availability

The data presented in this study are available on request from the corresponding author.

## Sample Availability

Samples of the compounds are not available from the authors.
